# Potential Role of Accessory Domains in Polyproteins Encoded by Retrotransposons in Anti-viral Defense of Host Cells

**DOI:** 10.3389/fmicb.2018.03193

**Published:** 2019-01-04

**Authors:** Sergey Y. Morozov, Alexander A. Lezzhov, Ekaterina A. Lazareva, Tatiana N. Erokhina, Andrey G. Solovyev

**Affiliations:** ^1^A. N. Belozersky Institute of Physico-Chemical Biology, Moscow State University, Moscow, Russia; ^2^Department of Virology, Biological Faculty, Moscow State University, Moscow, Russia; ^3^Faculty of Bioengineering and Bioinformatics, Moscow State University, Moscow, Russia; ^4^Shemyakin-Ovchinnikov Institute of Bioorganic Chemistry, Russian Academy of Science, Moscow, Russia; ^5^Institute of Molecular Medicine, Sechenov First Moscow State Medical University, Moscow, Russia

**Keywords:** anti-viral defense, retrotransposon, DNA transposon, RNA helicase, DNA methylase, insects, plants

Most autonomous retrotransposons and retroviruses encode, apart from the reverse transcriptase (RT), additional essential protein enzymatic domains including those of RNase H, protease and integrase, as well as a non-enzymatic gag-like domain (Eickbush and Jamburuthugoda, 2008; Arkhipova, 2017; Krupovic et al., 2018). Several types of accessory domains, the presence of which in retrotransposon polyproteins is non-stringent, are also reported. Particularly, well-known accessory domains in polyproteins of LTR-transposons include Chromodomain and DnaQ-like 3′-5′ exonuclease domain (Novikova et al., 2008; Rodriguez et al., 2017; Ustyantsev et al., 2017).

Previously we presented evidence for integration of the viral superfamily 1 RNA helicase (SF1 HEL or SF1H) coding sequences into insect genomes through acquisition by the retrotransposons containing no long terminal repeats (LTRs) (non-LTR-retrotransposons), namely, Long interspersed nuclear element-like (LINE-like) TRAS (Telomeric Repeat-Associated Element) of R1 clade in order Lepidoptera and LINEs of Jockey family in orders Hemiptera and Orthoptera. Moreover, in orders Diptera and Hymenoptera, the SF1 HEL domains were found to be translationally fused to proteins encoded by LTR retrotransposons (Lazareva et al., 2015; Morozov et al., 2017). These data were further confirmed and extended for the chromosome-integrated HEL sequences of plus-RNA viruses in orders Diptera, Lepidoptera, Hymenoptera, and Thysanoptera (Kondo et al., 2017; Geisler, 2018). Transposon-encoded helicases were found to contain the full set of conserved motifs essential for their enzymatic activities (Morozov et al., 2017) and exhibit a weak, but detectable, ability to suppress RNA silencing in plant experimental system, as it was previously demonstrated for RNA helicase domains of some replicative tobamovirus proteins (Csorba et al., 2007; Wang et al., 2012; Lazareva et al., 2015). Importantly, it is well-known that silencing suppressors of insect viruses are also active in plants (Maliogka et al., 2012). Moreover, although helicase-coding sequences represent actively transcribed insect genome regions, RNA helicase domains seem to perform no essential functions in retrotransposition and the transposon transcription/translation, and their functions can be considered as only accessory (Morozov et al., 2017).

We proposed hypothetic evolutionary scenarios explaining the natural selection-supported preservation of the retrotransposon SF1H domains in insect genomes and considered two basic alternatives to explain the long-term evolutionary fixation of SF1 HEL in retrotransposons, namely, significance of this genetic element as advantageous for (i) transposons themselves or (ii) their insect hosts (Morozov et al., 2017). In one scenario, we supposed that both siRNA- and piRNA-mediated pathways blocking expression and transposition of retroelements (Ito, 2012; Lazareva et al., 2015; Guida et al., 2016; Mondal et al., 2018) can be suppressed by the encoded SF1H silencing suppressor activity. Also SF1H-coding sequences acquired by retrotransposons might be adapted for direct co-operative work with reverse-transcribing enzymes to improve efficiency of cDNA synthesis and transposition of these selfish genetic elements (Morozov et al., 2017). Another scenario implied that genome-integrated RNA virus coding sequences producing virus-related transcripts and proteins may be a tool for anti-viral defense in plants, fungi, and animals (Honda and Tomonaga, 2016; Morozov et al., 2017; Palatini et al., 2017; Warner et al., 2018).

In recent years, a novel mechanism supporting the involvement of Dicer-encoded RNA helicases in anti-viral response in insects has been described in several pioneering works (Goic et al., 2013, 2016; Poirier et al., 2018). The structural basis for this activity is provided by the amino-terminal helicase Dicer domain that forms a clamp-like structure possessing several subdomains capable of binding both double-stranded and single-stranded RNAs and, likely, wrapping around RNA molecules. Moreover, Dicer can be stably bound to RNA without exerting the RNase III-like endonuclease activity (Song and Rossi, 2017). It was found that Drosophila and mosquito cells infected with ssRNA-containing viruses could produce cDNA fragments of RNA virus genomes by an endogenous reverse transcriptase activity, and that the resulting virus-specific DNA reinforced the host RNAi response against viral infections. Particularly, in Drosophila this mechanism was highly active in macrophage-like haemocytes (Tassetto et al., 2017). Most surprisingly, the virus-related cDNAs contained sequence junctions between LTR-retrotransposon and virus sequences derived from different genome parts (Goic et al., 2013). These studies provided a basis for a conceptually novel model of anti-virus response based on silencing, namely, production of secondary RNAi (Garcia-Ruiz et al., 2010; Pooggin, 2017) not deriving directly from genomic ssRNAs or their replicative forms (Goic et al., 2016; Tassetto et al., 2017). In a later work, it was shown that virus-related cDNAs produced during RNA-containing virus infection of insects included both linear and circular forms (Poirier et al., 2018). Circular DNA showed homology to both viral genomic sequences and LTR-retrotransposon sequences and participated in producing protective secondary siRNAs.

Considering the origin of DNA related to ssRNA viruses, it is important to note that the helicase domain of Dicer (Dcr-2) is crucial for biosynthesis of virus-specific DNA, and its activity is independent from the dicing function. Since RNA helicase domains of Dcr-2 (Poirier et al., 2018) or/and AGO (Tassetto et al., 2017) potentially recognize both retrotransposon RNA and viral dsRNA in the cytoplasm, it can be speculated that reverse transcription of viral RNA occurs because of the physical association of the RT complex and the dicing complex (Figure 1). Moreover, there is an indication that mostly minus-strands of viral RNA can serve as templates for reverse transcription (Poirier et al., 2018). From the evolutionary point of view, the described mechanism of integration of RNA virus-related circular DNA forms into host genomes may relate to origination of endogenous viral elements (EVEs) (Poirier et al., 2018), which are commonly associated with the invertebrate genomes (Holmes, 2011; Ballinger et al., 2012; Fort et al., 2012; Thézé et al., 2014; Metegnier et al., 2015; Geisler and Jarvis, 2016; Palatini et al., 2017; Suzuki et al., 2017) as well as with vertebrate chromosomes (Shi et al., 2018; Zhang et al., 2018).

**Figure 1:**
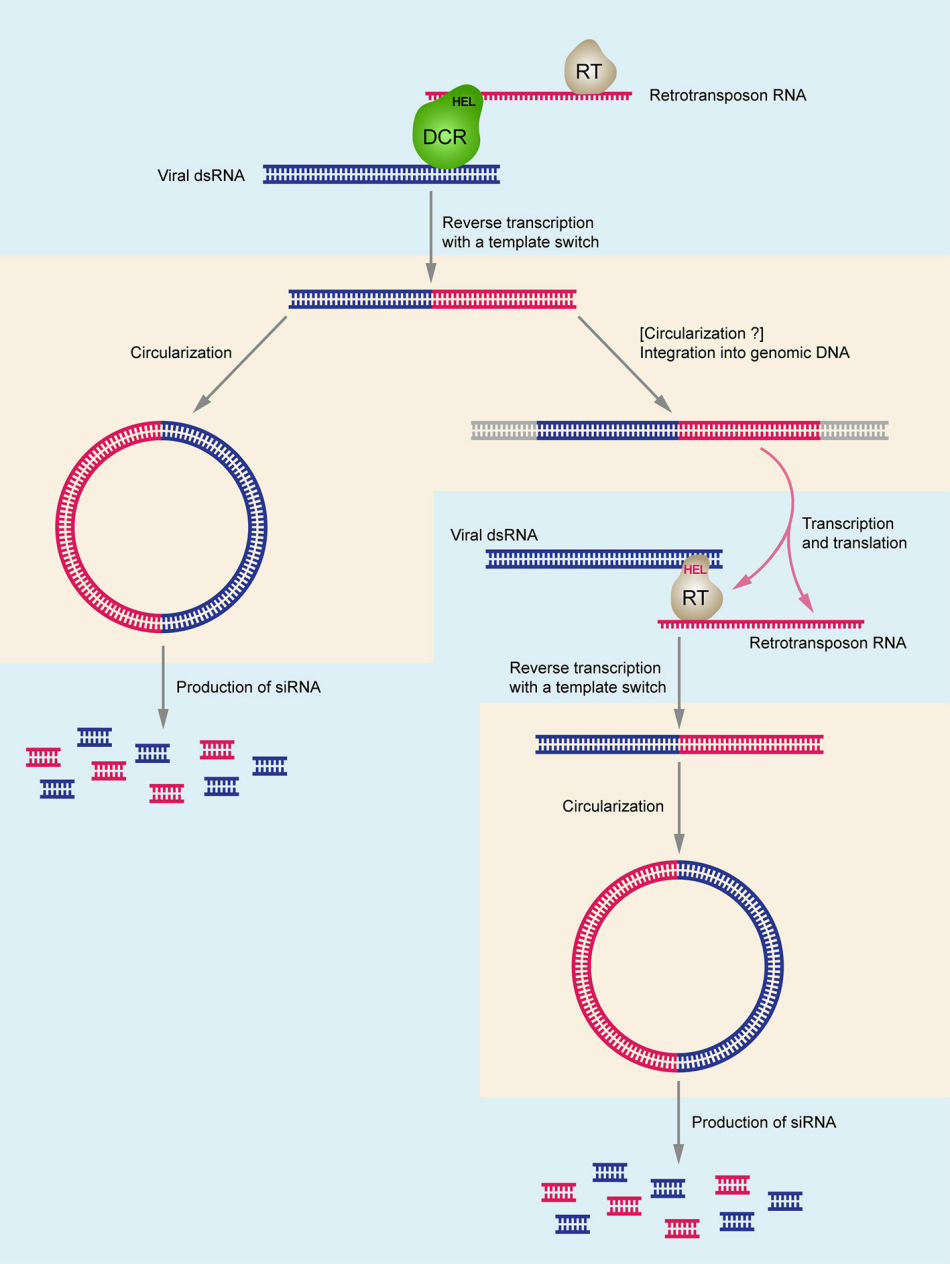
Schematic model of chimeric DNA synthesis and the interactions between DCR-HEL (RT-HEL), reverse-transcriptase (RT), retrotransposon DNA and viral dsRNA. Uptake of viral genomic RNA and synthesis of double-stranded replicative form RNA in cell cytoplasm stimulates chimeric circular and linear DNA production which allows increased *de novo* synthesis of virus-specific small RNAs and efficient antiviral RNAi response. Right part of the figure shows the hypothetical pathway specific for Lepidoptera insect species. DCR-HEL is shown in green; RT-HEL is shown in gray. Retrotransposon-specific RNA, DNA, and RNAi are shown in red; virus-specific RNA, DNA, and RNAi are shown in blue. DNA-specific steps are shown in pinkish areas; RNA-specific steps are shown in bluish areas.

The above results shed a new light on the phenomenon of viral SF1H domain acquisition by insect retrotransposon-encoded polypeptides described in our previous papers (Lazareva et al., 2015; Morozov et al., 2017). Indeed, enormous diversity of RNA viruses among many insect groups co-existing with their hosts for billions years of evolution (Dudas and Obbard, 2015; Li et al., 2015; Shi et al., 2016; Palatini et al., 2017; Bigot et al., 2018) suggests a demand for strong control mechanisms over infection processes. The abundant preservation of expressed SF1H in insect genomes could contribute to antiviral defense in some insect taxonomic groups. According to the hypothesis presented above, association of viral RNA helicase domain and reverse transcriptase domain in a single polyprotein or protein complexes can provide an effective mechanism for simultaneous reverse transcription of retrotransposon and viral RNA sequences into common cDNA molecules (Figure 1). Although initial experimental data have indicated the importance of LTR-transposons in the formation of RNA virus-related chimeric cDNA copies (Goic et al., 2013; Poirier et al., 2018), one can presume that non-LTR-retrotransposons are also well-suited for the process of chimeric cDNA synthesis from the RNA virus genomes and production of secondary virus-specific RNAi. Indeed, LINE transposons generate circular dsDNA products (Han and Shao, 2012) and contain internal promoters initiating synthesis of transcripts of both polarities from these products (Li et al., 2014; Russo et al., 2016).

Based on these ideas, we propose a speculative illustrative scheme for the evolutionary acquisition of SF1H domain by polyprotein of TRAS family LINE retrotransposons in Lepidoptera and its activity in anti-viral response (Figure 1). It is likely that the ancestor species of Lepidoptera contained abundant non-LTR retrotransposons of TRAS family that were transcribed and actively retrotransposed into the (TTAGG)*n* telomeric repeats to support the telomere length by repeat elongation (Fujiwara et al., 2005; Osanai-Futahashi and Fujiwara, 2011; Monti et al., 2013). Under conditions of high virus load, the RT complexes of these retrotransposons in association with RNA helicase domains of the cell Dicer and/or AGO enzymes (Goic et al., 2013; Poirier et al., 2018) can occasionally use the genomes of the (+)ssRNA viruses, which might be evolutionary close to Hubei-like viruses 1 and 2 (Shi et al., 2016; Morozov et al., 2017), to synthesize chimeric circular DNAs and transpose them into insect chromosomes. Those chimeric integrated transposon copies that encoded complete virus SF1 RNA helicase domains could be preserved in evolution because of their higher impact in anti-viral defense (Figure 1). The present-day Lepidoptera TRAS elements coding for SF1H domain obviously represent functionally specialized TRAS copies since they cannot be found in the vicinity of the (TTAGG)*n* telomeric repeats in contrast to copies containing no SF1H (Kondo et al., 2017; Geisler, 2018). Thus, Lepidoptera and many insect species belonging to other orders seem to gain efficient mechanism protecting the organism against a large variety of RNA-containing viruses.

Potential involvement of LINE retrotransposons encoding RNA helicases in anti-viral defense suggests that other defense genome elements can exist, possibly including different transposon types and different nucleic acid modifying enzymes. For example, for silencing-mediated pathogen protection, multiple (quite different) defense and counter-defense mechanisms were revealed (Pooggin, 2017). Indeed, it has become clear that bacteria also use reverse-transcribing elements for protection from DNA phages. These protective gene modules include, particularly, some CRISPR-Cas systems (Zimmerly and Wu, 2015; Koonin and Makarova, 2017). Strikingly, bacterial anti-phage AbiA and AbiK systems represent modules encoding a RT-like protein and a RecA-like SF1 DNA helicase (Scaltriti et al., 2011; Wang et al., 2011; Zimmerly and Wu, 2015) which is structurally related to viral SF1H (Gorbalenya et al., 1989). Moreover, bacteria and archaea are found to encode several types of multi-gene resistance modules (systems), including DNA helicase genes and some other genes (up to 4–5 cistrons). These modules include BREX system, DISARM system and Pgl system (Sumby and Smith, 2002; Barrangou and van der Oost, 2015; Goldfarb et al., 2015; Chaudhary, 2018; Ofir et al., 2018). Broad involvement of helicases in bacterial anti-viral defense systems suggests potential participation of additional enzymes targeting RNA/DNA as evolutionary selected protective tools. These enzymes could be involved in covalent modification of nucleic acids. In this respect, it is important that DNA methylase genes are the essential parts of the mentioned above anti-phage defense gene modules. Different types of these modules encode either an DNA N-6-adenine-methyltransferase (DAM) or C5 cytosine methyltransferase (DCM) (Barrangou and van der Oost, 2015; Goldfarb et al., 2015; Chaudhary, 2018; Ofir et al., 2018). The precise mechanisms of the anti-phage action of the above-mentioned DNA methylases (as well as helicases) are obscure. However, it is long known that some prokaryotic DNA methylases possess anti-phage activity and different phages are found to encode inhibitors of methylation (Krüger et al., 1989). Moreover, some bacterial transposons possess DNA methylase genes of the TnpB/Fanzor family (Bao and Jurka, 2013).

Strikingly, TnpB/Fanzor proteins were also encoded by several types of eukaryotic DNA transposons (Bao and Jurka, 2013). Moreover, DNA methylases are still encoded by eukaryotic retrotransposons, particularly, DAM protein domains were found as parts of polyproteins in DIRS elements (Goodwin and Poulter, 2001, 2004; Poulter and Butler, 2015; Kojima, 2018), and DCM-coding sequences were revealed in both Ty3/Gypsy and DIRS clades (de Mendoza et al., 2018). We speculate that some DNA methylases expressing as accessory protein domains from transposons may be involved in defense against DNA-containing viruses in eukaryotes like their specific prokaryotic counterparts (see above). DAM- and DCM-encoding retrotransposons of Ty3/Gypsy and DIRS clades were revealed in most Unikonts and some Bikonts (Rogozin et al., 2009), particularly, in Stramenopiles, Rhodophyta, green algae, and charophytes. Nevertheless, transposons encoding DNA methylases are not present in the genomes of land plants, such as tracheophytes (Goodwin and Poulter, 2001, 2004; Bao and Jurka, 2013; Szitenberg et al., 2014; de Mendoza et al., 2018). It is somewhat surprising that transposon-encoded methylases, which are found in many eukaryotes of Unikonta and Bikonta lineages (Rogozin et al., 2009), disappeared from the genomes of tracheophytes during land plant evolution. To our mind, disappearance of transposon-encoded methylases is connected to a great decrease in DNA virus abundance in land plants after evolving from algae, where large DNA viruses dominate (Correa et al., 2013; Middelboe and Brussaard, 2017; Weynberg et al., 2017; Schvarcz and Steward, 2018). Indeed, after evolving the land plants, the significance of DNA viruses for Viridiplanta became negligible because of inability of such viruses to infect land plant bodies (Dolja and Koonin, 2011), that made unnecessary the defense mechanisms against DNA viruses and resulted in evolutionary loss of transposon-encoded DNA methylases. However, anti-viral activity of non-transposon DNA methylases connected to transcriptional silencing still has a significant functional role in higher plants. It was shown that geminiviral Rep and C4 proteins were able to downregulate MET1 and CMT3 cell methyltransferases and prevent maintenance of *de novo* methylation at CG and CHG sites (Rodríguez-Negrete et al., 2013; Bräutigam and Cronk, 2018). Moreover, other gene products of geminiviruses (e.g., AC2) may influence methyl cycle of the host plant, particularly, affecting enzymes of the S-adenosylmethionine pathway (Yang et al., 2011; Zhang et al., 2011; Deuschle et al., 2016).

In conclusion, the presented hypothesis combines models for the mechanism of evolutionary origin and the functional role of retrotransposon-encoded nucleic acid-modifying domains, positioning these structural modules in the row of potential molecular tools for cell defense against viruses.

## Author Contributions

SM collected and analyzed the literature data, authored drafts of the paper. AL collected the literature data, prepared figure, reviewed the final draft. EL and TE collected and analyzed the literature data, reviewed the final draft. AS authored drafts of the paper, prepared figure, reviewed the final draft.

### Conflict of Interest Statement

The authors declare that the research was conducted in the absence of any commercial or financial relationships that could be construed as a potential conflict of interest.
